# Kinship Shapes Affiliative Social Networks but Not Aggression in Ring-Tailed Coatis

**DOI:** 10.1371/journal.pone.0037301

**Published:** 2012-05-18

**Authors:** Ben T. Hirsch, Margaret A. Stanton, Jesus E. Maldonado

**Affiliations:** 1 School of Environment and Natural Resources, Ohio State University, Columbus, Ohio, United States of America; 2 Smithsonian Tropical Research Institute, Panama City, Panama; 3 Center for Conservation and Evolutionary Genetics, Smithsonian Conservation Biology Institute, National Zoological Park, Washington, D.C., United States of America; 4 Department of Biology, Georgetown University, Washington, D.C., United States of America; 5 Department of Vertebrate Zoology, National Museum of Natural History, Smithsonian Institution, Washington, D.C., United States of America; University of Manitoba, Canada

## Abstract

Animal groups typically contain individuals with varying degrees of genetic relatedness, and this variation in kinship has a major influence on patterns of aggression and affiliative behaviors. This link between kinship and social behavior underlies socioecological models which have been developed to explain how and why different types of animal societies evolve. We tested if kinship and age-sex class homophily in two groups of ring-tailed coatis (*Nasua nasua*) predicted the network structure of three different social behaviors: 1) association, 2) grooming, and 3) aggression. Each group was studied during two consecutive years, resulting in four group-years available for analysis (total of 65 individuals). Association patterns were heavily influenced by agonistic interactions which typically occurred during feeding competition. Grooming networks were shaped by mother-offspring bonds, female-female social relationships, and a strong social attraction to adult males. Mother-offspring pairs were more likely to associate and groom each other, but relatedness had no effect on patterns of aggressive behavior. Additionally, kinship had little to no effect on coalitionary support during agonistic interactions. Adult females commonly came to the aid of juveniles during fights with other group members, but females often supported juveniles who were not their offspring (57% of coalitionary interactions). These patterns did not conform to predictions from socioecological models.

## Introduction

Kinship plays a key role in shaping animal societies, particularly in group-living species. In many societies, animals preferentially associate with kin, direct affiliative behaviors toward close relatives, and support close kin during agonistic interactions [1]. The role of kinship is thought to be particularly important in species that live in long-lasting, socially cohesive groups with sex-biased dispersal. In species that exhibit female philopatry, female relatives often support each other during agonistic interactions. Female dominance rank is greatly influenced by the number and dominance status of close kin [2]–[5], and dominance status can greatly influence stress hormone levels, food intake rates, longevity, and reproductive success [6]–[9]. Despite recent criticism of inclusive fitness models, kinship has been shown to be a major force shaping the evolution and structure of animal societies [10]–[12].

In addition to the key influence that genetic relatedness has on social organization, other factors including feeding ecology, competitive regime, and demography are also important. For example, socioecological models of resource distribution and feeding competition have been used to predict the presence and degree of nepotism in primate groups [13]–[14]. The inclusion of additional parameters such as the risk of infanticide and habitat saturation have created expanded socioecological models that can predict female grouping in gorillas and lions, and the formation of ‘friendships’ in chacma baboons (*Papio cynocephalus ursinus*) [15]–[19]. Within a social group, age, sex, social status, and hunger level can greatly influence an individual's choice of whom to interact with and in what manner [20]–[24]. Some species exhibit age-based homophily, in which individuals preferentially interact with conspecifics of the same age, regardless of relatedness [25]. Even temporary behavioral states, such as an individual's hunger level, have been found to change spatial association patterns and food related aggression [26]–[27]. Researchers have increasingly used social network analyses to address how these factors shape animal societies [28]–[29]. For example, Wey and Blumstein [23] used a network approach to determine that yellow-bellied “marmot colonies are largely organized based on age group and kinship.”

Ring-tailed coatis (*Nasua nasua*) are social carnivores that live in cohesive female philopatric groups. Unlike other coati species, each ring-tail coati group typically contains one adult male, except during the <1 month mating season, when several adult males can be found associating with each group [30]. Because coatis feed on contestable food resources and are female philopatric, we originally predicted that the ring-tailed coati social system would resemble primate groups with matrilineal dominance hierarchies [31]–[32]. However, the patterns of dominance observed in this species are apparently unique in that juveniles rank higher in the dominance hierarchy than adult females and subadults, which results in priority feeding access at fruit trees [33]. This does not appear to be related to patterns of ‘youngest ascendancy’ or ‘age-inversed dominance hierarchies’ found in some primate species [34]–[38]. Instead, almost all juveniles were ranked higher in the dominance hierarchy than all adults and subadults [32]. Given this atypical dominance hierarchy, it was suggested that maternal rank and kinship played little or no role in shaping the dominance rank of individual coatis [32]. These patterns contrast to previous work in coatis (*Nasua narica* and *N nasua*) that have demonstrated or strongly suggested that kinship plays a major role in shaping aggressive interactions and coalitionary support [39]–[40]. Further evidence suggests that adult females support non-offspring in dominance interactions, although this conclusion was based on maternity inferred from grooming behavior and not genetic data [32]. In addition, patterns of age-based homophily were found in regard to spatial association, and differences in rank appeared to influence particular age-sex classes to locate themselves in different within-group spatial positions [33], [41].

In the current study, we use social network analyses to determine the degree to which genetic relatedness influences the structure of three types of coati social networks: 1) spatial association, 2) grooming, and 3) aggressive interactions. Social network analysis allows for the quantification of multi-actor interactions, which provides a more realistic depiction of animal societies than traditional dyadic measures. With the use of these statistical and descriptive methods, it is possible to determine the degree to which kinship, age, and sex contribute to behavioral interactions between individuals and shape social structure. Although previous evidence suggested that kinship is not a major factor driving patterns of ring-tailed coati aggression, this hypothesis has never been explicitly tested. With the use of genetic markers we calculated relatedness between individuals and confirmed the identity of mother-offspring pairs. Here we test two non-mutually exclusive hypotheses: 1) Kinship explains the structure of coati social networks and 2) Age-sex class homophily explains the structure of coati social networks. Our predictions for each behavioral dimension are as follows:

### Association

#### Relatedness

Relatedness will be a significant predictor of association network structure. This pattern should hold between and among age and sex class categories.

#### Age-sex

Age and/or sex class homophily will be a significant predictor of the association network structure with higher association coefficients between members of the same age and sex class.

### Grooming

#### Relatedness

Relatedness will be a significant predictor of grooming network structure, with higher relatedness resulting in increased grooming rates. In particular, we predict that mother-offspring pairs should groom each other more frequently than more distantly related dyads.

#### Age-sex

Age and/or sex class homophily will be a significant predictor of the grooming network structure with more interactions between members of the same age and sex classes.

### Aggression

#### Relatedness

Relatedness will be a significant predictor of aggression network structure, with individuals directing less aggression towards related individuals [41].

#### Age-sex

Alternately, because coati dominance hierarchies have previously been reported to be ‘age structured’ we predict that relatedness could have little effect on aggressive networks [32] and age and/or sex class homophily will be a significant predictor of aggression network structure. Furthermore, if aggressive networks are primarily shaped by age and/or sex class homophily, we predict that particular age-sex classes will be more central in the dominance network structure, and give or receive more aggression than other age-sex classes.

Finally, because polyadic agonistic interactions can influence the structure of dyadic aggression networks, we conducted an additional analysis to determine the degree to which adult female coalitionary support for juveniles is shaped by kinship.

### Coalition formation

#### Relatedness

Adult females should preferentially support their offspring and/or closely related juveniles in agonistic interactions.

#### Age-sex

Adult females should support all juveniles during agonistic interactions, regardless of the degree of relatedness between the adult female and juvenile [32].

## Methods

### Ethics statement

This study complied with all institutional, national and ASAB / ABS guidelines for animal welfare. Local permission was granted from APN (Argentina National Park service) and animal handling procedures were approved by the SUNY Stony Brook Institutional Animal Use and Care Committee (IACUC# 20021175).

### Study site, subjects, and data collection

Behavioral data were collected in Iguazu National Park, Argentina (54°W, 26°S), between July 2002 and December 2004. A total of 150 coatis were captured in 32×10×12 inch Tomahawk or similar traps, immobilized with Ketamine and Xylazine and fitted with unique combinations of multicolored ear tags for individual identification (Rototag ear tags, Dalton Co.). Data from two neighboring coati groups (PQ and PSG) for two study years (2003 and 2004) were used in this study (N = 65 individuals). These two groups were socially segregated, and individuals rarely interacted with members of other groups. All coatis in the two social groups were individually recognizable due to their ear tags except for young juveniles which had not yet been tagged (juveniles were typically tagged when 4 months old). Coati groups were well habituated to the presence of human observers and we were able to follow habituated individuals within 2 m without disturbing them. Coati groups were comprised of adult females (24 months of age or older), subadults (12–24 months of age), juveniles (2–12 months of age), and one adult male (generally 36 months or older). Adult males disperse from their natal groups at 2 years of age, while females remain in their groups. Although group composition changed from year to year, group membership was relatively stable during the two study periods [41]–[42]. Any individual who died or dispersed during the study period was excluded from the analyses of that particular group-year (number of individuals included in the analyses for PSG 2003 = 12, PQ 2003 = 15, PSG 2004 = 25, PQ 2004 = 29). Most individuals changed age class from one year to the next, with the exception of the two adult males, 3 adult females in the PQ group, and 5 adult females in the PSG group.

All agonistic interactions were recorded ad libitum by the author, or by field assistants trained for at least 2 months [32]. Inter-observer reliability was tested during simultaneous observations of aggressive and grooming behavior, and field assistants were required to record interactions at ≥95% accuracy before their data were used. The winner of an interaction was defined following Gompper [43]: if one individual directed aggression towards a conspecific, and the recipient exhibited submissive behavior, the recipient was considered the loser. If an individual gained or maintained possession of a food item after an agonistic interaction, they were defined as the winner. A total of 1018 dyadic agonistic interactions were used in our analyses of aggressive behavior. These interactions included fights, chases, biting, lunges, aggressive vocalizations, displacements, and avoidances [32]. Dominance ranks were calculated using the MatMan program and results are presented in Hirsch [32]. Any interaction which involved more than two individuals was used in a separate analysis of coalitionary interactions [32]. Agonistic events were classified as a coalition when two individuals directed aggression at a third, or a third individual came to the aid of another during an agonistic event. A total of 37 coalitionary interactions involved an adult female aiding a juvenile. Grooming data were recorded ad libitum along with the identity of the individual grooming, recipient of grooming, or if the individuals were mutual grooming (total grooming bouts N = 1012). Grooming bouts were commonly observed during periods when the entire group would stop and rest in a safe location (such as a sleeping site or cliff edge). Almost all dominance interactions occurred during feeding and foraging (96.8% of occurrences) and aggression was particularly common when feeding on clumped fruit resources [32].

Ten second individual focal samples were recorded to determine levels of association between individuals [33], [41]. During a focal sample, the identity of all individuals within 3 m of the focal individual was recorded. This distance was chosen because the fruit species most commonly eaten by coatis (*Syagrus romanzoffianum*) has a fruit shadow of roughly 3 m radius [44]–[45], and thus this distance is biologically relevant for feeding competition and aggressive interactions sensu [46]. Relatively short focal samples were used because many of the associated variables recorded during the samples changed frequently (particularly the number and identity of neighbors within 3 m). Due to poor overall visibility in the dense forest, it was not feasible to select individuals based on a pre-determined order. Individuals were selected opportunistically, and the same focal individual was not resampled within ten minutes. Adults were preferentially targeted over juveniles, especially during 2004 when both groups had large numbers of juveniles. Due to a delay in trapping the PQ 2004 juveniles, a relatively low number of association scan samples were collected (N = 223), thus this group-year was excluded from the association network analyses (PQ 2003 N = 1306, PSG 2003 N = 1376, PSG 2004 N = 770).

### Genetic analyses

When individuals were captured, a small plug of skin tissue was punched out during ear tagging and the tissue was stored in 10% DMSO saline solution. DNA purification was carried out using a Qiagen Bio-Sprint 96 workstation following the protocol for DNA extraction from animal tissues as supplied by the manufacturer. All individuals were genotyped at 15 previously developed microsatellite loci which averaged 4.2 alleles per locus (range 2–7) [47]–[51]. Optimized PCR temperatures and reaction conditions for all loci are detailed in Hirsch and Maldonado [52]. Products were electrophoresed through an ABI 3130xl genetic analyzer and fragment size analysis was performed using the GeneMapper software (Applied Biosystems, Inc., Foster City, CA). All samples were amplified and genotyped at least two times for each locus. We used the program CERVUS 3.0 to determine mother-offspring pairs [52]–[53] and *Relatedness* 5.0 [54] to calculate pairwise relatedness based on the allele frequencies of all adult individuals in the population. We found no evidence for null alleles or linkage disequilibrium in our population [52].

### Social networks

Three social networks were built for each group for each year based on matrices of: 1) association, 2) grooming interactions, and 3) aggressive interactions. In the association network a connection, or tie, existed between any two individuals who were observed associated as defined above. These ties were weighted based on that dyad's halfweight coefficient, which is a commonly used measure of association. The halfweight coefficient is essentially a corrected ratio that accounts for differences in sighting frequency or sampling effort by comparing the number of times individuals were seen together to the number of times they were seen in total and is calculated as X/X+0.5(Ya+Yb)+Yab where X = number of times individuals a and b were observed together, Ya is the number of observations where a was observed without b, Yb is the number of observations in which b was observed without a, and Yab is the number of observations a and b were both observed, but in separate groups [55]. All association-based social networks were undirected. Grooming networks were built from grooming interaction data and ties were weighted based on the number of grooming events that occurred between dyads. Grooming networks were directed such that ties were outgoing from the actor and incoming to the recipient. Aggression networks were also directed and constructed similar to grooming networks using the results of agonistic interactions. Halfweight coefficients were calculated in SocProg 2.4 [56].

### Statistical analyses

We used multiple regression quadratic assignment procedures (MRQAP) with the double semi-partialing permutation method to determine what factors influenced social structure in the coati groups [57]. The MRQAP is an extension of the Mantel test which allows for a dependent matrix to be regressed against multiple independent matrices [58]. We conducted three separate MRQAPs with association, grooming, and aggressive interactions as the dependent matrices, while age-based homophily, sex-based homophily, genetic relatedness, and mother-offspring pair as the independent matrices. For the homophily matrices, similar dyads received a value of 1, while dissimilar dyads received a value of 0. In the mother-offspring matrices, adult female and juvenile offspring pairs received a value of 1, while all other dyads were coded as 0 s. Mother-subadult offspring were coded as 0 in this matrix. MRQAP regressions were run in UCINET 6.3 [59].

We investigated general differences between age-sex classes with respect to grooming and aggression network measures. Three measures of centrality were calculated for each individual in each network: in-strength, out-strength, and eigenvector centrality. In-strength centrality (also known as weighted in-degree) is defined as the sum of the weights of all incoming ties, where as out-strength (weighted out-degree) is the sum of the weights of all outgoing ties. Eigenvector centrality is the corresponding eigenvalue of the first eigenvector of a given matrix and is a measure of both direct and indirect connectedness [29], [60]. Centrality measures were normalized based on group size to facilitate comparison between networks. All network centrality metrics were calculated using the igraph package for R 2.13 [61]–[62].

Patterns of coalitionary support were assessed by comparing the number of observed cases of mother-offspring coalitionary support to the predicted number if females randomly aided all juveniles. We also tested whether adult females preferentially supported closely related juveniles (i.e. not just offspring). We compared the average degree of relatedness between juveniles and the adult females that supported them to the average pair-wise relatedness of social group members using ANOVA tests carried out in JMP 5.1.

## Results

### MRQAP Regressions

The mother-offspring matrices were significant predictors of the association and grooming networks in all group-years (Table 1). Genetic relatedness was significantly related to grooming and association patterns in almost all group-years, but only when mother-offspring matrices were not included in the MRQAP regressions. The addition of a matrix of same-age maternal siblings (i.e. nestmates) did not yield significant results in relation to grooming, association, or dominance in any of the four group-years. The MRQAP regressions did not reveal significant, directionally consistent sex-based homophily in any network. Age-class homophily, however, was a significant predictor of association and grooming patterns in some cases. Juvenile homophily was a significant predictor of association (all Pvalues<0.002), while subadult homophily was a significant predictor in one group-year and trended in the same direction in another group-year (PQ 2003 P = 0.075, PSG 2004 P = 0.001). Interestingly, there was no effect of relatedness or mother-offspring pairs on dyadic aggressive behavior (Table 1).

**Table 1:** MRQAP regression results for association, grooming, and aggression networks in two social groups (PQ and PSG) during 2003 and 2004.

Groups:	PQ 2003		PQ 2004		PSG 2003		PSG 2004	
Association	slope	P	slope	P	slope	P	slope	P
Mother-offspring ***	0.338	0.002*	-	-	0.207	0.005*	0.241	0.001*
Relatedness	0.044	0.325	-	-	0.068	0.216	0.033	0.292
Sex	0.014	0.374	-	-	0.009	0.430	0.084	0.043*
Adult	0.071	0.205	-	-	0.014	0.433	0.013	0.420
Subadult	0.127	0.075	-	-	-	-	0.205	0.001*
Juvenile ***	0.682	0.002*	-	-	0.835	0.001*	0.372	0.001*
**Grooming**								
Mother-offspring ***	0.568	0.001*	0.689	0.001*	0.569	0.001*	0.527	0.001*
Relatedness	0.063	0.179	−0.012	0.298	−0.017	0.440	−0.031	0.208
Sex	0.034	0.283	0.009	0.324	0.081	0.179	0.056	0.054
Adult ***	0.077	0.125	0.226	0.001*	0.252	0.007*	0.390	0.001*
Subadult	0.121	0.049*	-	-	-	-	0.027	0.134
Juvenile	−0.129	0.028*	−0.037	0.071	−0.020	0.421	−0.065	0.088
**Aggression**								
Mother-offspring	−0.069	0.191	−0.024	0.260	−0.024	0.424	−0.026	0.334
Relatedness	0.058	0.233	−0.012	0.407	0.095	0.248	0.047	0.175
Sex	0.150	0.004*	−0.064	0.076	−0.221	0.015*	0.046	0.110
Adult	−0.014	0.490	0.064	0.073*	0.017	0.419	−0.013	0.455
Subadult	−0.066	0.049*	-	-	-	-	0.003	0.377
Juvenile	−0.070	0.246	−0.024	0.357	0.116	0.184	−0.052	0.268

Age class categories represent age homophily. No subadults were present in the PQ 2004 and PSG 2003 groups. Significant predictor variables (P<0.05) for individual group-years = *. Variables that were significant in at least three out of four group years = ***.

### Grooming network metrics

**Figure 1:**
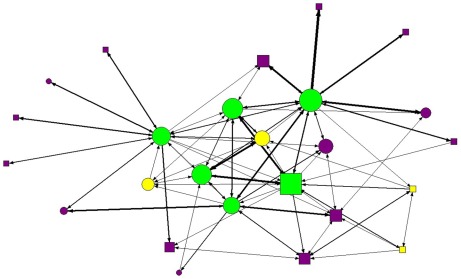
Grooming interaction network from the PSG 2004 group. The nodes represent individual coatis and the thickness of the lines between nodes is proportionate to the number of interactions between those individuals. Circles: females; squares: males; green: adults; yellow: subadults; purple: juveniles.

Adult females groomed each other more often than predicted (in three out of four group-years) but this age homophily pattern was not seen in the grooming behavior of juveniles and subadults (Table 1). Adult females groomed others more than all other age-sex classes, while adult males received the most grooming (Figure 1, Table 2).

**Table 2:** Average normalized grooming network eigenvector, out-strength, and in-strength values for each age-sex class ±standard deviation.

Age-class	N	Eigenvector	Out degree strength	In degree strength
Adult female	10	0.622±0.288	89.497±64.325	44.305±27.843
Adult male	2	0.549±0.203	35.874±19.543	74.471±53.047
Subadult female	4	0.373±0.159	35.224±21.114	21.448±11.069
Subadult male	2	0.080±0.010	9.653±2.275	4.826±2.275
Juvenile female	25	0.224±0.169	6.188±10.380	22.061±27.605
Juvenile male	28	0.236±0.179	11.709±14.909	23.387±21.371

N = number of individuals. Adult females groomed others the most, while adult males received the most grooming. Values were averaged across groups and years.

### Aggression network metrics

**Figure 2:**
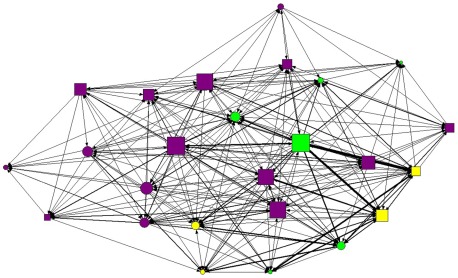
Aggressive interaction network from the PSG 2004 group. The nodes represent individual coatis and the thickness of the lines between nodes is proportionate to the number of interactions between those individuals. Circles: females; squares: males; green: adults; yellow: subadults; purple: juveniles.

Males were more central in the aggression network than females (average eigenvector centrality males = 52.974±25.128 SD, females = 23.839±15.698) and directed more aggression than females (average normalized out strength degree males = 47.134±52.683, females = 16.910±22.192). Adult males were particularly aggressive, and directed more aggression than other age-sex classes (Figure 2, Table 3). Male juveniles also had higher aggression out-strength values than juvenile females (males = 41.087±43.901, females = 13.906±13.351, Table 3).

**Table 3:** Average aggression network eigenvector, out-strength, and in-strength values for each age-sex class ±standard deviation.

Age-class	N	Eigenvector	Out degree strength	In degree strength
Adult female	10	0.431±0.190	22.419±31.460	42.066±30.989
Adult male	2	0.685±0.373	105.399±87.312	26.820±28.192
Subadult female	4	0.440±0.373	10.859±14.522	63.935±70.177
Subadult male	2	0.739±0.004	15.255±6.780	39.226±3.698
Juvenile female	25	0.266±0.144	13.906±13.351	19.989±23.071
Juvenile male	28	0.423±0.251	41.087±43.901	24.957±26.723

N = number of individuals. Adult and juvenile males directed the most aggression to others, while adult females and subadults received the most aggression. Values were averaged across groups and years.

### Coalitions

A total of 37 cases of adult female coalitionary support of juveniles were recorded in the two main study groups during 2003–2004. If adult females (3–5 per group-year) randomly gave support to all juveniles, it was expected that juveniles would be supported by their mother in 8 incidences. We found that mothers supported their offspring twice as much as random (16 cases), but a larger proportion of adult female support for juveniles was from non-mothers (57%). No between group differences were found in the proportion of cases where adult females supported their offspring (PQ = 42%, PSG = 44%). Adult females also did not preferentially support closely related juveniles; the average degree of relatedness between juveniles and the adult females that supported them was not statistically different from the average degree of relatedness between individuals in the social group (ANOVA tests; PQ: F_1,798_ = 0.028, P = 0.866; PSG: F_1,647_ = 0.057, P = 0.391).

### Group relatedness

**Figure 3:**
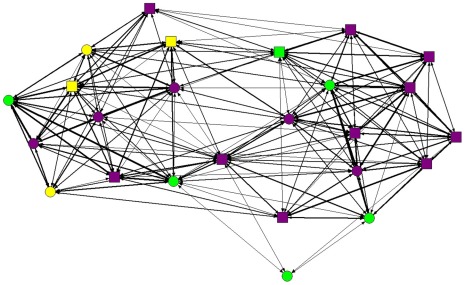
Aggressive interaction network from the PSG 2004 group. The nodes represent individual coatis and the thickness of the lines between nodes is proportionate to the degree of relatedness between those individuals. The bottommost adult female is JW, who was not closely related to the other adult group members. Circles: females; squares: males; green: adults; yellow: subadults; purple: juveniles.

The two study groups varied in the degree to which individual coatis were related to each other, which likely arose from the distinct origins of the two groups. The PQ group was founded by a single adult female and her offspring in 2001, while the PSG group formed when five adult females split off from a larger group in late 2002 [63]. Adult females in the PQ group were more closely related to each other than adult females in the PSG group (average pairwise relatedness ±SD: PQ = 0.191±0.247, PSG = −0.020±0.299, P = 0.02). Because all PQ group members were the offspring of the founder adult female, pairwise relatedness in the PQ group was higher than in the PSG group (Figure 3; average pairwise relatedness, PQ = 0.126±0.237, PSG = 0.034±0.279, P<0.001). One female in the PSG group (JW) was more distantly related to all other adult females (average pairwise relatedness to other adult females = −0.146±0.133), despite the fact that no observed instances of adult female immigration or emigration were observed during the 2.5 year study period. The other four PSG adult females may have been pairs of sisters or other close female relatives (pairwise relatedness PS-GH = 0.693, NY-CM = 0.253).

## Discussion

Grooming and association matrices were shaped by mother-offspring associative behavior in all four group-years. This result demonstrates the strong link between genetic relatedness and associative behaviors in ring-tailed coatis. Interestingly, pairwise relatedness values were not a significant predictor of grooming and association when mother-offspring pairs were included in the MRQAP regressions. While juveniles were more often associated with other juveniles, they did not preferentially associate with their closest juvenile relatives (i.e. same age maternal siblings). These results are consistent with the idea that affiliative behaviors in coatis are strongly shaped by mother-offspring relations, while other kinship categories are less important (full and half siblings, aunts, etc.).

In general, age class homophily was a weak predictor of grooming network structure. Subadults and juveniles rarely groomed within their age class, but grooming among adults was a significant variable determining grooming network structure in three out of four group-years (Table 1). Adult females frequently groomed others, including juveniles, other adult females, and the adult male (Figure 1). The adult male in each group received more grooming than other age-sex classes (Table 2). This pattern was likely related to a behavior in which several coatis would simultaneously approach the adult male and groom him together or in succession. This behavior occurred most frequently when the adult male rejoined the group after briefly leaving (typically for 0.25–12 hours). We suspect that these greeting behaviors may function to reinforce social bonds (cf. [64]).

Adult females frequently groomed each other, even though foraging adult females were not always within close proximity. These patterns contrast strongly with juveniles and subadults who rarely groomed within their age class, but generally associated with their same age class during foraging [33], [41]. We posit that the association and grooming matrices are measuring two different aspects of affiliative behavior. Grooming behavior is likely a better measure of social bonding, whereas spatial associations may be primarily shaped by socio-ecological factors such as predation and feeding competition. Individuals of different body size, foraging needs, and dominance status may choose, or be forced into, different within-group spatial positions (reviewed in: [65]–[66]). Similar patterns of age and size assortative behavior have been found in many other vertebrate species (fish-[21], [67], ungulates-[68], primates-[69]–[71]). A previous study of these coati groups found that juveniles preferred to be at the front edge of the group to arrive first at quickly depleted fruit trees, while subadults were forced to the group margins by aggressive adult females [33], [41], [43]. It appears that the proximity matrices are largely being shaped by feeding competition and aggression, while grooming patterns are shaped by mother-offspring bonds, female-female social relationships, and a strong social attraction to adult males.

Aggression network structure was not explained by kinship, or mother-offspring pairs. Indeed, it appears that none of the tested parameters reliably predicted the structure of aggression networks. Although no age or sex class homophily variables were significant predictors of the overall dominance network structure, a closer comparison of the direction of interactions within and between age-sex classes demonstrated clear patterns. Not surprisingly, adult males were particularly aggressive (Figure 2). It has been posited that adult males who are better at fighting and chasing away other adult males are preferred by adult females [52]. Similarly, juvenile males were generally more aggressive than juvenile females. If these aggressive experiences during early social development lead to greater fighting ability as adults, increased juvenile male aggression could have an adaptive function [72]. Subadults received more aggression than all other age-sex classes. This behavior has previously been linked to coalitionary support for juveniles by adult females [32]. Typically, when a juvenile has an agonistic encounter with a subadult, one of the adult females in the group will come to the aid of the juvenile and violently chase the subadult, even when the adult female is the mother of the subadult. It is this support by adult females which allows juveniles to direct aggression towards subadults with little fear of serious reprisals.

The patterns of coalitionary support between adult females and juveniles are only marginally consistent with the hypothesis that adult females preferentially support their offspring during aggressive interactions. Juveniles were supported by adult females that were not their mothers during more than half of these coalitionary interactions (57%). It is plausible that patterns of coalitionary support found in ring-tailed coatis could have arisen due to inclusive fitness benefits, with adult females supporting closely related juveniles in addition to their own offspring. In the PQ group, where all adult females were closely related to all juveniles, inclusive fitness benefits could have easily led adult females to support all juveniles. On the other hand, there was variability in the degree of relatedness among group members. Even if most individuals were closely related, it was predicted that females should preferentially support their offspring during aggressive conflicts with their siblings, parents, and aunts. We found little evidence to support these patterns. In the PSG group, adult females were more distantly related to each other than females in the PQ group. The one adult female (JW) which was more distantly related to all other adult females still came to the aid of non-offspring juveniles (N = 5). These patterns indicate that close kinship is not necessarily a prerequisite for coalitionary aid in this species. Our result that relatedness had no discernable effect on patterns of aggression is in stark contrast to most studies of aggression and dominance in social animals [1], [5], [73]–[75]. To our knowledge, this is the first example where genetic relatedness has little or no influence on agonistic behavior in a highly social mammal (for an avian example, see: [76]).

In some species, group augmentation has been posited as a hypothesis for female tolerance of juvenile aggressive behavior. Clutton-Brock and colleagues [77]–[79] found that larger meerkat groups are able to outcompete smaller groups, have lower costs of raising offspring, lower mortality, and higher breeding success. These authors concluded that meerkats likely aid young juveniles to augment group size, thus resulting in higher fitness levels for older group members. In our coati study population, we found no evidence that an increase in group size had a beneficial effect on the above factors, thus it does not appear to be a plausible reason for juvenile dominance in ring-tailed coatis [32].

The unusual age-based aggression patterns in ring-tailed coatis appear to fall outside the purview of widely used socio-ecological and kinship based models of animal behavior. No previously published model of animal behavior would have predicted that adult females should direct aggression towards subadults regardless of their kinship ties, while coming to the aid of non-offspring juveniles. This coalitionary support provided by adult females is the major reason why juvenile coatis were able to feed in small patchy resources without being excluded by larger individuals, and thus provided a major fitness benefit to the youngest, most vulnerable age-class. Although previous studies have documented adult females preferentially aiding younger offspring in other species [7], [80], we know of no example in which adult females direct aggression towards older offspring in the defense of younger, non-offspring. We posit that although kinship based models of social aggression are widely applicable to a large number of animals, not all social mammals conform to their predictions, and studying these exceptions in further detail could lead to better future models predicting patterns of social aggression.
